# The Children's Ward as a Special Department

**Published:** 1912-07-27

**Authors:** 


### THE MAKING OF A MODERN HOSPITAL.

### The Children's Ward as a Special Department.

The children's ward should be considered as a special departmental unit or station, worked and administered as all such units are in co-operation with the rest of the units that constitute the hospital. It is now generally considered advisable that it should be placed in charge of a special member of the medical staff, known as the Physician-in-Charge of the Children's Department, who must be a specialist in pediatrics. But the corollary that the surgical work in connection with the ward must be undertaken by a member of the surgical staff who has specialised in diseases of children is not so generally accepted; it is still customary to call in the surgeons indiscriminately when an operation has to be done, and it may, therefore, happen that cases belonging to various members of the surgical staff are housed in the ward. This, to a certain extent, destroys the special character of the ward. It is far better to have one surgeon attached to the ward, and to let him work in close co-operation with the Physician-in-Charge; in this way the best results, undoubtedly, are obtained.

Similarly with regard to the nursing staff. The nurse attached to the ward must have special knowledge of children's diseases and their treatment; if she has had her training in a children's hospital so much the better, but if she has been trained in a general hospital she must undergo a period of probation in the nursing of children's diseases before she is given charge of the beds in the children's ward. The nursing of children demands special qualifications, almost a special aptitude, and a nurse who has been accustomed solely to adult nursing usually fails to manage a children's ward in the way it should be managed. Attention to these administrative details will be found to pay in the end, mainly in shortening the stay of the patients in the ward. The head nurse or sister in charge of the ward should have served a special apprenticeship in a children's hospital.

The ward annexes demand some particular attention. We have already outlined the special points in the construction of closets and lavatories, and pass on to the bathrooms. The need for a moveable bath, which can be wheeled to the bedside, is less apparent in this ward than in the ward for adults. Sick children are easily carried by a single nurse, and it is generally better to bathe them in the bathroom attached to the ward. The baths here should be of china or strong enamelled earthenware; a copper bath looks very attractive and lasts well—so long as it is kept scrupulously clean; to keep it spick and span, however, entails considerable trouble and labour, and it will generally be found that a china bath is in every way much more suitable and satisfactory. A frequent mistake is to make the children's baths similar in size and shape to those employed in the wards for adults, whereas they should be smaller, shallower, and narrower. Needless to say the baths should be so placed that they are quite separated from the adjoining walls and give free access from all points to the attendant nurse. The children should not be allowed to enter the bathrooms unattended. Attention to the ventilation and heating of the bathroom should be one of the first considerations of the constructor.

### The "Barrier" System.

An important adjunct to the children's ward is the isolation room. This, indeed, is indispensable, and it is better to have two or more isolation rooms than to depend on one alone for the segregation of doubtful cases. Children are very susceptible to infectious diseases, and child patients are often admitted to a ward under conditions which render it necessary that they should be carefully watched. Scarlet fever and chicken-pox are, perhaps, the most common diseases which in the children's ward are likely to infect the other patients; whooping-cough is another and very dangerous enemy which may enter unexpectedly; mumps and measles are also insidious in their entry to the ward. To depend on the "barrier" system in a children's ward is to court disaster; the only efficient safeguard is the isolation room in which a suspect case can be observed for a few days until its nature is definitely cleared up, when it can be removed either to a fever hospital or isolation ward in case it proves to be a case of infectious disease, or placed in the ward in case it proves to be free from infection.

Where should Isolation Rooms be Placed?

The isolation rooms must, therefore, be placed in such a position that there is no risk of carrying infection from them into the general ward. Various methods have been employed, most of which are compromises and cannot honestly be said to be ideal. In some wards the isolation rooms are placed close to the entrance, on one side of a general lobby which gives access to the main ward; in others they are placed in juxtaposition to the annexes at the farther end of the ward. In the former arrangement every entrant to the ward has to traverse the danger zone; an obviously unfortunate necessity which seems calculated to interfere with the due prevention of the spread of infection. In the second arrangement the patients have to be carried through the ward to reach the isolation rooms, while the nurses who are in charge of the cases have to go through the ward in coming from or going to their charges. Where the isolation room is placed close to the general annexes it is equally difficult to guard against infection and to secure that absolute segregation which is indispensable.

For these reasons it has become more generally realised that it is better to have the isolation rooms absolutely distinct and shut off from the general ward, and to allocate to them a small side ward with a separate entrance and distinct bath and lavatory rooms. Such an isolation annexe is preferably placed at the farther end of the ward, separated from the general end annexes by a dividing corridor, with entrances to the general ward and into an entrance lobby, where suspect patients are received. The ward entrance must be so arranged that it can be conveniently shut off in case of necessity, so that all admittance to the isolation annexe is obtained through the outside entrance. A couple of moderately sized rooms, each containing two beds and a cot, will generally be found sufficient. Every institution has its own method of calculating the percentage of isolation cases it may have to deal with; as an average minimum it may be taken that one isolation bed or cot for every six beds or cots is a fair estimate for a children's ward. The general arrangement and furnishing of the isolation room will be dealt with in a special article of this series.

In hospitals where each unit or station is housed in a separate pavilion it is easy enough to secure conditions which approach as closely as possible to the ideal. In older institutions, especially in those in which the children's ward is an adapted ward, it is generally very difficult to secure conditions which are even approximate to the ideal. Much may be done, in these latter cases, by reconstruction, and by replanning so as to eliminate certain features of the general ward which are unnecessary in the children's ward. One of these is the day room. If this is needless in a ward destined for adult patients, it is still less necessary in a children's ward. On the other hand, ample balcony or outdoor verandah accommodation is desirable, and such accommodation is usually more easily secured for the children's than for the ordinary wards, since the outdoor space wanted is less in the case of child patients. A good, well guarded balcony is the best day room attached to a children's ward. It should be so arranged that its floor level is equal to that of the ward floor, so that the cots or beds can easily be wheeled out. It must have adequate protection from rain and (a point which is sometimes entirely overlooked) from the wind as well. Wind protection is best afforded by simple screens which can be removed when necessary. If the ward is on the ground floor care should be taken to guard the patients from outside annoyances by a fairly high protective railing.

The Glass Balcony.

In newer hospitals on the Continent effective protection is secured by enclosing the verandah or balcony with clear glass screens; this is excellent on the ground floor, but objectionable if the balcony is on a higher level, unless wire netting or iron railings are added as a safeguard in case of accidents. The children may be allowed to have their meals served on the balcony, and should be encouraged to be in the open air as much as possible. If a day room is absolutely necessary, a part of the ward itself, screened off from the rest, may be utilised, but, generally speaking, a day room is unnecessary in a children's ward. It must be remembered that as the ward is a unit, it should, as such, be complete in itself.

We would lay stress, therefore, on the desirability of providing a proper ward laboratory, a dressing room, and a ward kitchen. The dressing room is often overlooked. In how many children's wards do we find that ordinary surgical procedures, involved in the after-treatment of operated cases, are carried out with merely the semi-privacy obtainable by use of a few ward screens. Children are much more susceptible to pain and to sensory impressions than adults, and where such procedures are accompanied by pain the dressings very often lead to general disturbance and annoyance in the ward. The patients scream, and by so doing affect the other children, who sometimes join in sympathetic chorus. To perform these ordinary dressings, such as syringing a sinus, removing empyema tubes, or packing cavities with gauze, in the ward is indefensible in a hospital that claims to be up to date and modern in the best sense of the term. A proper dressing room must be provided.

